# Shared decision‐making for older adults with cardiovascular disease

**DOI:** 10.1002/clc.23267

**Published:** 2019-10-03

**Authors:** Warren D. Backman, Sharon A. Levine, Nanette K. Wenger, John Gordon Harold

**Affiliations:** ^1^ Veterans Affairs New England Geriatric Research Education and Clinical Center Boston Massachusetts; ^2^ Section of Geriatrics Boston University and Boston Medical Center Boston Massachusetts; ^3^ Division of Palliative Care and Geriatric Medicine Massachusetts General Hospital Boston Massachusetts; ^4^ Division of Cardiology Emory University School of Medicine Atlanta Georgia; ^5^ Emory Heart and Vascular Center Atlanta Georgia; ^6^ Emory Women's Heart Center Atlanta Georgia; ^7^ Cedars‐Sinai Smidt Heart Institute and UCLA David Geffen School of Medicine Los Angeles California

**Keywords:** aged, cardiovascular disease, clinical decision‐making, decision aids, decision‐making capacity, elderly, geriatric cardiology, geriatrics, informed decision‐making, older adults, patient centered care, shared decision‐making

## Abstract

Shared decision‐making is appropriate for clinical decisions involving multiple reasonable options, which occur frequently in the cardiovascular care of older adults. The process includes the communication of relevant factual information between the patient and the clinician, elicitation of patient preferences, and a mutual agreement on the best course of action to meet the patient's personal goals. For older adults, there are common challenges and considerations with regard to shared decision‐making, some of which (eg, cognitive impairment) may be biologically linked to cardiovascular disease. There are tools designed to facilitate the shared decision‐making process, known as decision aids, which are broadly effective although have shortcomings when applied to older adults. Novel approaches in clinical research and health systems changes will go some way toward improving shared decision‐making for older adults, but the greatest scope for improvement may be within the grass roots areas of communication skills, interdisciplinary teamwork, and simply asking our patients what matters most.

## 1 CLINICAL VIGNETTES

Case Vignette 1: Born in 1919, the patient was educated at the Curtis School of music with his classmate Leonard Bernstein. He was a violinist and music arranger who served as concertmaster at the Hollywood Bowl under Leopold Stokowski. As a classically trained pianist his musical legacy included working with The Eagles, The Beach Boys, Neil Diamond, and many others. The patient had known severe aortic stenosis but had deferred intervention until a syncopal episode led to an acute care hospitalization. The patient developed troponin elevation and chest pain. He underwent coronary angiography but following coronary cannulation he developed cardiogenic shock. The patient had emergency aortic balloon valvuloplasty and coronary stent deployment and stabilized. Subsequent shared decision‐making focused on whether to proceed with transcatheter aortic valve replacement (TAVR). The patient was quite functional at 92 and opted to proceed. He developed renal failure post TAVR. He continued to write musical scores and coproduce music until he died at age 94. He reported good quality of life post TAVR despite becoming dialysis dependent.

Case Vignette 2: A 95‐year‐old woman was transferred from an outside hospital due to worsening heart failure related to critical aortic stenosis, which was her only medical problem. She had been transferred to the current hospital with a view to TAVR as her only option for therapy. Her family was very eager for her to undergo the procedure to “save her life.” She and her family met with the house staff, general cardiology, and TAVR teams. At the meeting, she stated that her most pressing priority was to return to her home, her plants, and her sun room. She understood her condition and the benefits and risks of TAVR. She was discharged with home hospice care.

## 2 BACKGROUND

Shared decision‐making has been lauded as the paragon of clinical decision‐making, but what exactly does it mean in theory and in practice? Why should we do it? Are we not already doing it? What are the challenges when applying it to the cardiovascular care of older adults?

### 2.1 What is shared decision‐making?

There are different approaches to clinical decision‐making. The traditional approach, often termed the *paternalistic* approach, involves the clinician deciding which course of action is best, and then presenting this course of action to the patient. The flow of expertise is unidirectional, and even if patients are well informed, their involvement is limited to their right not to give consent or, depending on the scenario, not to adhere to the recommendation. Although the term *paternalistic* is sometimes used pejoratively, this type of decision‐making is appropriate in situations where there is only one reasonable course of action. For example, consider an otherwise healthy 60‐year‐old man, who takes no medications, smokes cigarettes, and presents to the clinic with stable typical angina pectoris. The only reasonable course of action as an initial step is to recommend smoking cessation and to initiate appropriate medical therapy. It would be absurd to suggest that the patient, after discussing his preferences with the clinician, might leave the clinic having reached a mutual decision with the clinician to forego any medication and to continue smoking.

However, many clinical situations are not straightforward and entail multiple reasonable options from which to choose. In these situations, the culture of modern medicine is moving away from the paternalistic model of decision‐making, to one of shared decision‐making. In shared decision‐making, the flow of expertise is bidirectional; the clinician provides clinical expertise and the patient provides expertise regarding their personal situation, values, preferences, and attitude toward risk. Rather than the clinician deciding what's best for the patient, instead they discuss the options and agree together upon the best one. According to commonly used terminology, the decision is said to be *shared*.

There are several published descriptions of what constitutes good shared decision‐making^1^, ^2^ most of which can be distilled to the following three essential ingredients:

1. Provision of evidence‐based information about reasonable options, tailored to the individual patient
2. Deliberate elicitation of patient preferences
3. Decision support according to the patient's needs, which may include the use of decision aids or other forms of counseling

### 2.2 The move toward shared decision‐making

The gradual paradigm shift in modern healthcare away from paternalism and toward shared decision‐making has been edging along for decades. In 1982 in the USA, the President's Commission on ethical issues in medicine produced a chapter entitled “Informed Consent as Active, Shared Decisionmaking.”^3^ The notion of shared decision‐making was later encapsulated by the phrase “nothing about me, without me,” as articulated by Valerie Billingham in her seminar Through the Patient's Eyes in Salzburg, 1998.^4^ Later refined to “no decision about me, without me,” the phrase was adopted as a battle cry by those making the case that shared decision‐making should be the norm.^1^ Although the original focus was on informed consent, shared decision‐making also encompasses medication management, self‐care, and lifestyle changes. Shared decision‐making is the clinical interaction at the core of patient‐centered care, meaning “care that is respectful of and responsive to individual patient preferences, needs and values,” which was identified by The Institute of Medicine in 2001 as a key goal for improving the quality of healthcare in the USA.^5^ It has been enshrined into law, including the 2010 Patient Protection and Affordable Care Act Sec 3506 which sets out “to facilitate collaborative processes… that engages the patient, caregiver or authorized representative in decision‐making…and facilitates the incorporation of patient preferences and values into the medical plan.”^6^

The perceived need for shared decision‐making may be even stronger nowadays. The internet and boom of technology have made huge amounts of health‐related information freely accessible to an extent unimaginable a few decades ago. This accessibility of information has to some degree tipped the balance of knowledge a little less toward physicians and a little more toward the patients. Patients overall are increasingly more empowered to be active in clinical decision‐making and may enlighten clinicians with the findings of their own research. An approach that relies on the “doctor knows best” mantra may seem to patients to be passé or even demeaning. On the other hand, misleading or wholly inaccurate information is prevalent online and in other media; this magnifies the need for clinicians to assess patients' knowledge, ideas, and expectations and to help them navigate all available information before arriving at medical decisions.

Indeed, the implementation of shared decision‐making is gathering steam as a priority in many healthcare systems internationally, including in the United Kingdom where it “is a key component of Universal Personalised Care” which is part of the National Health Service Long Term Plan^7^ and in Canada where The Canadian Task Force on Preventive Health Care has set out the role for shared decision‐making in preventive healthcare.^8^

As it pertains to the practice of cardiology in the USA, the authors are aware of four decisions by the Centers for Medicare and Medicaid Services for cardiovascular procedures that require a shared decision‐making encounter as a condition of reimbursement: Left atrial appendage closure therapy,^9^ ventricular assist device implantation,^10^ TAVR,^11^ and implantable cardioverter defibrillator (ICD) placement.^12^

### 2.3 Counterarguments to shared decision‐making

As the saying goes, the road to hell is paved with good intentions. Therefore, efforts to revolutionize healthcare, no matter their virtue, are deserving of scrutiny; shared decision‐making is no exception. While shared decision‐making has been described as an ethical imperative,^1^ the soundness of the concept has been challenged on semantic and philosophical bases.

The term “shared decision” has been called a misnomer. McNutt^13^ argued that only a patient knows whether the intended outcome of a treatment is worth accepting potential adverse effects, and therefore “shared decision‐making cannot exist.” Rather, he argues, the term “informed medical decision‐making” might be a better term as it is information, not a decision, that is shared.

In a separate critique of the current model of shared decision‐making, Gillick^14^ points out that while it makes sense in principle, it is not working in practice due to poor adherence to it among physicians. She therefore suggests “re‐engineering” shared decision‐making so that patients are not asked to decide on a specific treatment but rather to communicate their goals of care and physicians then help to translate those goals into actual medical care. This approach “alters the division of labor between patients and physicians. It assigns the values component to the patient and the technical component to the physician without fundamentally altering the concept of patient engagement.”

The above arguments are entirely valid, but really address tensions that need not exist. One tension arises when there is too much focus on the precise mechanics of how a decision is made rather than the factors that support its making. Another arises when there is dogmatism about how forcefully patients should be pushed to be decisive. In our interpretation, shared decision‐making as described widely in the literature is really a potpourri of complementary processes involving not only the decision itself, but also the encircling aspects of the clinician‐patient relationship. Exactly how these processes are employed and to what extent will vary depending on the individual patient, as any patient centered approach should.

## 3 ELICITING GOALS OF CARE

To attempt to make a shared medical decision without knowing a patient's goal of care is like trying to choose a mode of transportation without reference to a destination. Sometimes the goal of care is self‐evident and does not need to be elicited. For example, if a patient is reporting frequent bothersome palpitations due to an atrioventricular nodal reentrant tachycardia, then the goal of care is to prevent the palpitations. The decision‐making would revolve around the options of medication vs an ablation procedure, and further options within either approach.

Oftentimes, however, the goal is not self‐evident. Consider again the patients in the vignettes above, both of whom had severe aortic stenosis. In one case the patient's goal was to continue his musical passion and to live longer. In the other case, the patient's goal was to die peacefully at home.

It is important to be mindful that older adults, particularly those with multiple chronic conditions, may have a very different set of care goals compared to younger, healthier adults (see Figure 2). There may be less focus on survival and more focus on quality of life, preserving independence, and function.

**Figure 1:**
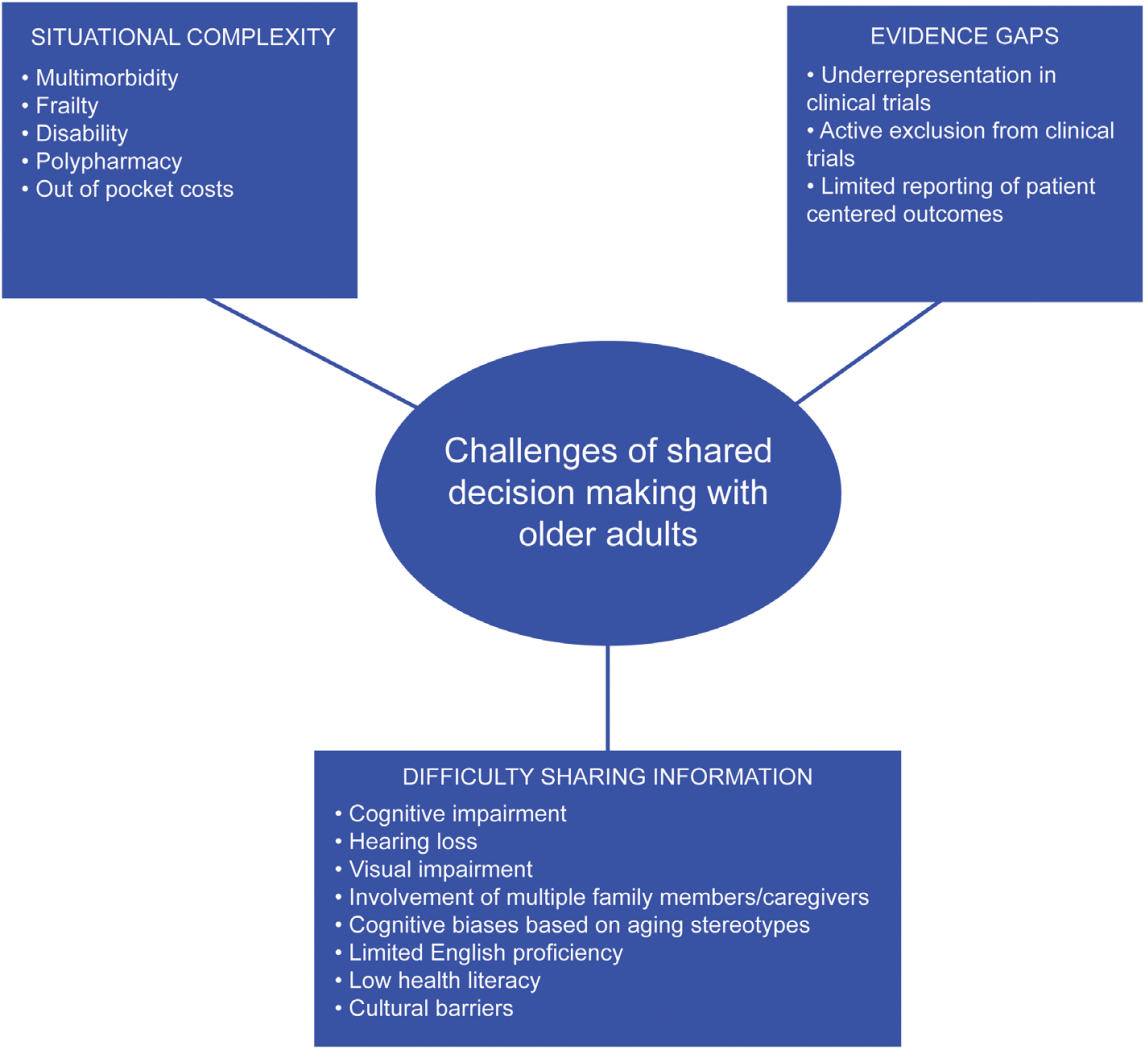
Challenges of shared decision making with older adults. These can be conceptualized in three distinct categories: (a) difficulty sharing information, (b) evidence gaps, (c) situational complexity

**Figure 2:**
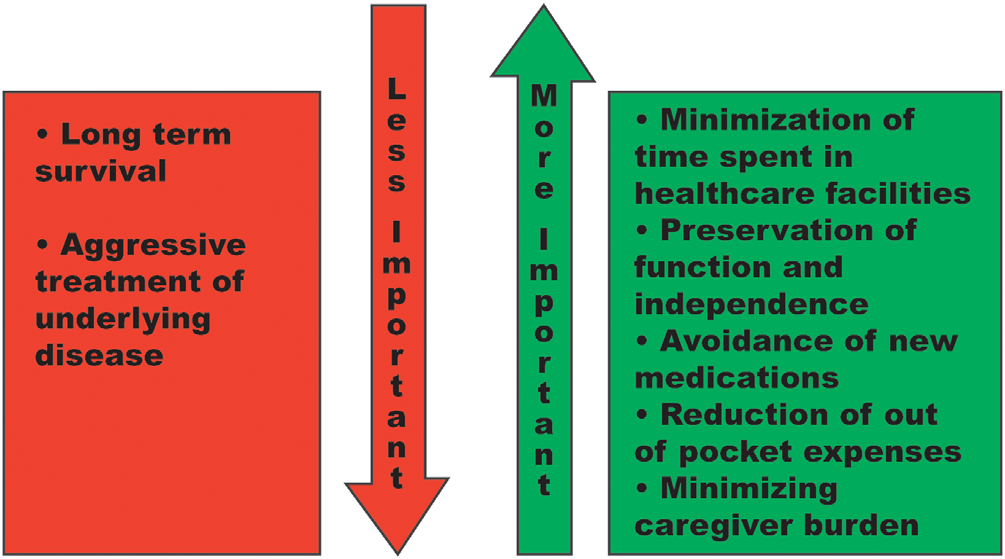
Relative importance of health goals may change with age. Although assumptions should not be made about an individual patient's goals of care, this figure serves as an example of how certain goals may become of increasing importance as time goes by, while others may be of decreasing importance

Assessment of goals of care is just as crucial in the outpatient setting as it is in when patients are critically ill. Some phrases that can be useful when trying to elicit a patient's goals are as follows

- “what do you think is most important when considering your future health?”
- “what would you be willing to sacrifice to gain a few more years/months? What would you not be willing to sacrifice?”
- “is there anything that would be against your wishes if you became critically ill?”

Clinicians also have to be certain that stable patients do not perceive a routine request to elucidate their goals as a tacit indication that something ominous has occurred or is imminently to occur. The best way to avoid this misunderstanding is by stating clearly upfront that it is not the intention.

## 4 CHALLENGES OF SHARED DECISION‐MAKING WITH OLDER ADULTS

Time is often cited as the principal impediment to shared decision‐making.^15^ This is particularly true for older patients who tend to have multiple cardiovascular issues, not to mention other medical issues, competing for attention at any given clinical encounter. However, even if ample time is available, there are many other challenges to implementing shared decision‐making with older adults (Figure 1). Older adults comprise a much more heterogeneous population than their younger counterparts, with a remarkable spectrum between those who are highly functional, robust, and independent to those who suffer much chronic disease and require total care from others. In discussing challenges of shared decision‐making in older adults, the intention is not to generalize, but rather to be cognizant of common issues that may arise.

### 4.1 The challenges of sharing medical information with older adults

Information is the universal currency of shared decision‐making but it has no value unless it can be given, received, and processed. Many things can get in the way of this.

Cognitive impairment is a formidable challenge in this arena. When it is present but does not affect one's ability to perform essential everyday activities, the term *mild cognitive impairment* is used. *Dementia* is the term used when cognitive impairment does impact such activities. Cognitive impairment overall increases in prevalence with age and is often undiagnosed, with one study showing that physicians are unaware of cognitive impairment in 40% of cases and mostly do not document mild cognitive impairment even when it is recognized.^16^ Of relevance to cardiology, vascular disease and hypertension are causally linked with cognitive impairment via microvascular brain damage,^17^, ^18^ and heart failure is associated with it as well.^19^ Cognitive impairment may be obvious when interacting with a patient, but not necessarily. Some patients may have developed ways of consciously or subconsciously hiding their impairment in the course of conversation; examples being nodding or using vague comments like “okay” to give the impression of understanding, changing the subject of conversation, making a joke instead of answering a question, or using phrases like “you're the doctor, you tell me” or “my [caregiver] handles that.” Even the subtlest impairment can affect the processing and retention of information that is directly relevant to medical care. This can have a knock‐on effect on decision‐making capacity, which is discussed below. There is insufficient evidence to support routine screening for cognitive impairment in older adults, but it may be helpful in cases where the sharing of information is proceeding with more difficulty than expected. A brief simple written and verbal test such as the Mini‐Cog, which can be done in under 3 minutes, is helpful to objectively assess the likelihood of cognitive impairment and identifies patients who may warrant more detailed testing for confirmation.^20^

Even if the brain is working fine, the ears may not be. Hearing loss is very common. Disabling hearing loss is present in 25% of adults aged 65‐74, rising to over 50% above 75 years old.^21^ Cardiovascular disease itself, and individual risk factors for cardiovascular disease, are associated with hearing loss particularly in older adults,^22^, ^23^ and there may be causative links.^24^ As with cognitive impairment, patients can mask their hearing loss. Furthermore, hearing loss can be mistaken for cognitive impairment. Most of the communication in clinical encounters is given verbally and so care must be taken by the clinician to ensure that the patient has heard what was said. Patients may be embarrassed if they keep having to ask the clinician to repeat themselves, and so may simply say nothing. Noisy inpatient wards with beeping cardiac monitors may make it particularly hard for the patient to hear conversation. If the patient with hearing loss is accompanied by an involved relative, friend, or other caregiver, it is all too easy for them to fade into the background of the conversation. It is no surprise then that evidence has shown that hearing loss is associated with decreased satisfaction with clinician‐patient communication and quality of care,^25^ as well as decreased knowledge, skills, and confidence to participate actively in healthcare.^26^ Where hearing loss is impeding clinical discussion, and hearing aids are not available or functional, the use of a relatively inexpensive personal sound amplification product^27^ can be enormously helpful. These generally use headphones and can be kept in the clinic for use by consecutive patients provided that appropriate hygiene measures are taken.

On a similar note, visual impairment is also more prevalent among older adults and shares risk factors with heart disease.^28^ Over the age of 65, 6.6% of adults have visual disability.^29^ While this does not interfere with verbal communication, it certainly can interfere with sharing of written or graphical information pertaining to medical decisions.

Aside from pathologic impediments to communication, cognitive biases based on stereotypes of old age can distort the sharing of information related to medical decisions. Older adults may wrongly attribute symptoms of disease to normal aging and therefore resign themselves to live with these symptoms when they could be solved, decreased, or mitigated with medical care. Clinicians may make the same errors and neglect to present reasonable avenues of diagnosis or treatment that would be offered to younger patients. Other examples include assuming that older adults do not want to be actively involved in decision‐making which can be a self‐fulfilling assumption, leading to inappropriate paternalism. As a rule of thumb, if it seems that a decision is being made based purely or predominantly on chronological age, it is time to step back and examine the decision critically.

As of 2013, the number of people in the USA with limited English proficiency was estimated to be 25.1 million, 15% of whom were over the age of 65.^30^ The convenience of using an accompanying friend or family member to interpret during clinical counters is alluring. However, this is highly discouraged as the accuracy of the translation is questionable, and these individuals may deliberately withhold certain information from the patient or the clinician, based on good or bad intentions. A telephone interpretation service could be a better option, but it is limited by hearing ability and audio quality. The best option, where available, is to have a professional interpreter in person. The visual cues from gestures and lipreading can help overcome hearing impairment, and the interpreter can more easily develop a rapport with the patient that supports the shared decision‐making process.

Additionally, beyond language itself, cultural behaviors may lead older adults deliberately to defer to their younger family members and/or to the clinician; particularly when it comes to racial and ethnic minorities.^31^ This is an example of where a dogmatic approach to shared decision‐making is inappropriate. Patients cannot and should not be forced to engage in the decision‐making more than they want to do, but clinicians should still try to gently explore the patient's goals and concerns to the greatest extent afforded. Professional interpreters can also be helpful to provide insight into cultural dynamics.

All these challenges with information sharing may manifest at a macroscopic level as low health literacy. Health literacy is defined by the World Health Organization as “the cognitive and social skills which determine the motivation and ability of individuals to gain access to, understand and use information in ways which promote and maintain good health.”^32^ Older age has been identified as a risk factor for low health literacy, particularly for those aged 85 or above.^33^, ^34^

The involvement of multiple family members and/or caregivers is another feature sometimes seen with older patients; it is a blessing and a curse. Older patients in some cases have one, two, or even three generations of family members involved with their care, as well as other paid or voluntary caregivers. Generally, they provide crucial collateral information, as well as invaluable psychosocial and practical support to the patient. On the other hand, they can have their own perceptions, or even agendas, which do not always align with the patient's. In the worst‐case scenario, caregivers may have ulterior motives, such as financial in nature, which may underlie their input into a medical decision. Furthermore, at a practical level, clinical encounters can take longer to make decisions with more people involved; sometimes multiple visits and/or telephone calls are needed to involve all the key stakeholders.

### 4.2 The challenges posed by gaps in evidence

Our earlier definition of shared decision‐making requires evidence‐based information tailored to the individual. That assumes the availability of relevant evidence. Older adults have generally been underrepresented in cardiovascular clinical trials or deliberately excluded based on age cutoffs.^35^

Even when cardiovascular clinical trials do include older adults, they may not include participants with multiple coexisting chronic conditions. Among adults over 80 years of age, multimorbidity is more common than any single disease, and just over half have four or more chronic conditions.^36^ The older adult sitting in front of you likely does not resemble those in clinical trials.

The evidence base is even thinner when it comes to patient centered outcomes such as measurement of symptoms, function, or quality of life. Instead clinical trials have largely focused on clinical events such as mortality, sudden cardiac death, or target vessel revascularization.^37^ There is a move to include more patient‐centered outcomes in clinical trials, but there is a long way to go until the evidence base is as robust as it is for traditional clinical outcomes.

One could argue that shared decision‐making is dead in the water without numerical evidence available to support clinical decisions, as indeed has been opined.^13^ However, given that the decision does not make itself, it seems that the clinician has to attempt to engender a shared decision by presenting the best available evidence, even if it is wholly anecdotal.

### 4.3 The challenges posed by situational complexity

So far, we have discussed general challenges with sharing information, and the lack of information to share from clinical trials. The third category of challenge in shared decision‐making with older adults relates to the milieu in which the decision is made, hereafter termed situational complexity. Simply put, there can be so many medical and social factors to consider, that it is hard to know which clinical options are reasonable to explore with the patient.

Multimorbidity is the norm^36^ and layered on top of this are the closely related but separate phenomena of disability and frailty, the latter being defined as “a state of increased vulnerability to poor resolution of homeostasis following a stress.”^38^

Cognitive impairment, as well as interfering with the sharing of information, may have a bearing on the clinical options proposed. First, there is the issue of practicality; for example it is generally unwise to offer a medication that requires multiple daily doses to somebody with memory problems, even if there is a prognostic benefit in clinical trials. Second, cognitive impairment has prognostic implications that may affect a decision to proceed with an intervention, for example, if it is associated with postoperative delirium.^39^ Given that cognitive impairment is so often undiagnosed,^16^ it should be actively identified where it would alter the risk‐benefit relationship. As mentioned earlier, brief screening tools are available.

Social circumstances which are relevant to the medical decision at hand may not be apparent during a clinic visit or hospital admission. In the USA, above the age of 65, 7.8% of older adults have a disability that affects self‐care and 14.2% have a disability that affects independent living. Above the age of 75, these figures rise to 13.1% and 23.9%, respectively.^40^ For older adults with disability, there is an interplay between function and social factors such as home environment, support from others, and finances. The effects of this interplay may manifest in scenarios such as medication nonadherence and frequent hospital readmissions. They also impact rehabilitation, for example after heart failure admissions or cardiac procedures.

The out‐of‐pocket costs for medications for older adults in the USA are substantial and are noteworthy when making shared decisions about initiation of new medications. As of 2015, for example, heart failure was among the top 20 conditions entailing the greatest percentage of Medicare Part D enrollees with stand‐alone prescription drug plans with high out of pocket drug costs. Of patients with heart failure, 8.7% were classified as having high out of pocket costs, with an average expense of $2971 per year.^41^ The addition of costly medications might be enough to tip a patient toward hardship, and furthermore the cost of medication is associated with nonadherence in heart failure.^42^ Financial factors may therefore heavily weigh into a decision.

With so many situational factors to consider, it can be hard for the clinician and patient to make decisions. This is where a multidisciplinary approach is key. The decision can be shared with other members of the multidisciplinary team. Consider enlisting the help of colleagues from one or more disciplines such as primary care, geriatrics, palliative care, nursing, social work, physical/occupational therapy, pharmacy, or nutrition.

In the outpatient setting, multidisciplinary case conferences may be helpful, akin to multidisciplinary cancer conferences. There is some precedent for this in cardiology, with one such program demonstrated at New York University (NYU) Langone Health/NYU School of Medicine.^43^

For decisions with very high stakes, in the ideal scenario multiple team members meet with the patient, family, and other close caregivers. While this tends to happen in extreme scenarios during hospitalizations, it is hard to schedule such meetings in the outpatient setting for practical reasons. However, a scientific statement from the American Heart Association suggested an annual heart failure review, much like an annual wellness visit in primary care, as an opportunity to revisit goals.^44^ If such an annual review was conducted, it seems that it would be best done with multidisciplinary input. Although it may be more facile to assemble the appropriate team during a hospitalization, the ideal venue is during a more stable phase of the disease in an outpatient setting.

On a final note about situational complexity, with so many moving parts the scenario is often in a state of flux, and decisions may need to be reframed as situations change. For example, consider a patient with heart failure, osteoporosis, and gait instability, who considers their quality of life to be acceptable and values longevity. A decision may be made to have an ICD inserted on an elective basis for primary prophylaxis of sudden cardiac death. However, in the interim the patient may fracture their hip leading to an admission complicated by delirium and significant functional decline and self‐reported diminished quality of life. The decision to implant an ICD may no longer be appropriate and should be revisited. However, if the patient subsequently rehabilitates well and regains what they consider to be an acceptable quality of life, the decision to place an ICD is again reframed.

## 5 DECISION‐MAKING CAPACITY

Assessing a patient's decision‐making capacity is a critical component of shared decision‐making, especially for patients who may have some cognitive impairment or mental illness. This approach includes four components,^45^ which are based on the ethical principle of autonomy or the right to self‐determination. The patient must be able *to express* choice. The patient must *understand* the diagnosis, prognosis, proposed treatment options, and risks and benefits of those treatments as well as the risks and benefits of declining various treatment options. In addition, the patient must be able *to appreciate* the diagnostic and treatment options and possible outcomes in their own case. Finally, the patient must be able *to apply reasoning* in comparing various treatment options. For example, in the second vignette, the patient must be able to state that she does not want to undergo TAVR but would rather go home with medical management. She should be able to demonstrate her knowledge of the prognosis and outcomes on her symptoms and disease progression with and without TAVR. She must *appreciate* the likely outcome of continued symptoms of heart failure, other symptoms, and death by choosing to forego the procedure but opting instead to die in her own home surrounded by family with a palliative approach to symptom management. There are decision tools to aid clinicians in asking questions in a way that will elicit responses that can get at the answers to these four components.^46^ There is a “sliding scale” for decision‐making: high risk procedures or treatments require a higher standard of capacity; low risk treatments require a lower standard.

## 6 DECISION AIDS

Decision aids are evidence‐based devices to facilitate shared decision‐making and may take the form of pamphlets, videos, or interactive tools which might be available via the internet.^47^ Decision aids are appealing because they harness data from epidemiologic studies and clinical trials to make them applicable to the individual. They make statistics more digestible and can be done at home at the patient's convenience. The most recent Cochrane review of decision aids as recent as 2015, included 105 studies with over 31 000 participants. Twelve (11%) of the 105 studies were of decision aids for cardiovascular issues, a curiously low number considering the relative burden of cardiovascular disease and the relatively large evidence base for cardiovascular medicine.

Key results from the Cochrane review are that when people use decision aids, they improve their knowledge of the options, the benefits and harms of each, and feel better informed and clearer about what matters most to them. They probably participate more in decision‐making with clinicians.^48^

There have been other clinical trials of decision aids for cardiovascular care since 2015, for example DECIDE‐LVAD.^49^ There are also decision aids that can be helpful but have not necessarily been studied in clinical trials. The American College of Cardiology's CardioSmart website, for example, provides several decision aids covering the management of atrial fibrillation, aortic stenosis, and heart failure.^50^

Given all the above, it is tempting to conclude that decision aids should be used routinely in the cardiovascular care of older adults. However, there are several reasons to be circumspect.

Decision aids themselves are rooted in the evidence base, which is lacking for older adults as we have described. The number of decision aids put to trial in older adults is vanishingly small. A 2016 systematic review is enlightening in this respect.^51^ It only included studies that evaluated participants with a mean age of 65 years or older or reported on a subgroup of participants aged 65 or older. After reviewing 11 000 references, only 22 studies met the criteria for inclusion. Only one decision aid was specifically developed for older adults and the mean age in most studies was between 65 and 70, capturing only the youngest of older adults. Only seven of those 22 studies related to cardiovascular issues, mostly atrial fibrillation.

Ironically, while too little of an evidence base can make it difficult to develop decision aids, a rapid expansion of the evidence base (such as the one called for in older adults) may make it hard for decision aids to keep pace with emerging evidence, leading them to become quickly outdated^52^, ^53^ especially where funding for further development dries up.

Even the best decision aid for older adults may be inherently limited due to its inability to consider the whole picture including the complex relationship between multiple chronic diseases, function, and social circumstances. They may not be useable for people with visual, hearing, or cognitive impairment, limited English proficiency or low (health) literacy.

To quote another perspective, when decision aids are developed for older adults for use at home, “it is not always obvious how much of their time patients should spend reviewing information and completing questionnaires instead of pursuing their lives and loves.”^15^

Notwithstanding the above limitations, decision aids when used judiciously in the cardiovascular care of some older adults, may be vital lubrication for the shared decision‐making process.

## 7 FUTURE DIRECTIONS

There remain many questions about how best to incorporate shared decision‐making into routine clinical care in the broadest sense. This will rely on a combination of evidence to support best practice, systems change directed at the health policy level,^54^ and a continued cultural shift.

To further enhance shared decision‐making in the cardiovascular care of older adults, there is a need for more clinical trials that include older adults with and without multimorbidity, and that report more patient centered outcomes. Data from such trials will catalyze the development of decision aids specifically geared toward older adults facing decisions in their cardiovascular care.

## 8 CONCLUSION

Older adults with cardiovascular disease, and the clinicians caring for them, often face difficult choices between multiple reasonable options. Shared decision‐making enables both parties to contribute their differing but equally important areas of expertise to find the best path toward the patient's personal care goals. Such an endeavor is overall more nuanced in an older adult. It may take longer, and many considerations must be made with respect to aging‐related issues to avoid pitfalls in the process. Novel approaches in clinical research and health systems changes will go some way toward improving shared decision‐making for older adults, but the greatest scope for improvement may be within the grass roots areas of communication skills, interdisciplinary teamwork, and simply asking our patients what matters most.

“For age is opportunity no less Than youth itself, though in another dress, And as the evening twilight fades away The sky is filled with stars, invisible by day.”

Henry Wadsworth Longfellow

## CONFLICT OF INTEREST

The contents do not represent the views of the U.S. Department of Veterans Affairs or the United States Government.
